# Integrated transcriptomics and metabolomics analysis provides insights into aromatic volatiles formation in *Cinnamomum cassia* bark at different harvesting times

**DOI:** 10.1186/s12870-024-04754-w

**Published:** 2024-02-02

**Authors:** Shaochang Yao, Xiaoming Tan, Ding Huang, Linshuang Li, Jianhua Chen, Ruhong Ming, Rongshao Huang, Chun Yao

**Affiliations:** 1 College of Pharmacy, Guangxi University of Chinese Medicine, Nanning, 530200 China; 2 Key Laboratory of Protection and Utilization of Traditional Chinese Medicine and Ethnic Medicine Resources, Education Department of Guangxi Zhuang Autonomous Region, Nanning, 530200 China; 3 Guangxi Scientific Experimental Center of Traditional Chinese Medicine, Guangxi University of Chinese Medicine, Nanning, 530200 China

**Keywords:** *C. cassia*, Cinnamon, Aromatic volatiles, Transcriptomic and metabolomic analysis, Regulatory network

## Abstract

**Background:**

*Cinnamomum cassia* Presl, classified in the Lauraceae family, is widely used as a spice, but also in medicine, cosmetics, and food. Aroma is an important factor affecting the medicinal and flavoring properties of *C. cassia*, and is mainly determined by volatile organic compounds (VOCs); however, little is known about the composition of aromatic VOCs in *C. cassia* and their potential molecular regulatory mechanisms. Here, integrated transcriptomic and volatile metabolomic analyses were employed to provide insights into the formation regularity of aromatic VOCs in *C. cassia* bark at five different harvesting times.

**Results:**

The bark thickness and volatile oil content were significantly increased along with the development of the bark. A total of 724 differentially accumulated volatiles (DAVs) were identified in the bark samples, most of which were terpenoids. Venn analysis of the top 100 VOCs in each period showed that twenty-eight aromatic VOCs were significantly accumulated in different harvesting times. The most abundant VOC, cinnamaldehyde, peaked at 120 months after planting (MAP) and dominated the aroma qualities. Five terpenoids, α-copaene, β-bourbonene, α-cubebene, α-funebrene, and δ-cadinene, that peaked at 240 MAP could also be important in creating *C. cassia*’s characteristic aroma. A list of 43,412 differentially expressed genes (DEGs) involved in the biosynthetic pathways of aromatic VOCs were identified, including phenylpropanoids, mevalonic acid (MVA) and methylerythritol phosphate (MEP). A gene-metabolite regulatory network for terpenoid and phenylpropanoid metabolism was constructed to show the key candidate structural genes and transcription factors involved in the biosynthesis of terpenoids and phenylpropanoids.

**Conclusions:**

The results of our research revealed the composition and changes of aromatic VOCs in *C. cassia* bark at different harvesting stages, differentiated the characteristic aroma components of cinnamon, and illuminated the molecular mechanism of aroma formation. These foundational results will provide technical guidance for the quality breeding of *C. cassia*.

**Supplementary Information:**

The online version contains supplementary material available at 10.1186/s12870-024-04754-w.

## Background

*Cinnamomum cassia* Presl, commonly known as Chinese cinnamon, belongs to the Lauraceae family and is a major food spice and traditional Chinese medicine with important commercial prospects. *C. cassia* is widely distributed in southeast Asia, Indonesia, and South America [1], but is mainly grown in the southern provinces of China, Guangxi, Guangdong, and Yunnan, where its cultivated area is more than 270,000 ha [2]. The bark of *C. cassia* is most frequently used in Western countries as a spice and seasoning, but as a drug in Asian countries. In China, it is a common traditional Chinese medicine. The Pharmacopoeia of the People’s Republic of China (CH.P) includes *cinnamomi cortex* (Rougui) in more than 500 formulas for its anti-inflammatory, analgesic, and stomachic properties [3, 4]. So far, over 160 chemical compounds have been identified from *C. cassia*, with a wide range of pharmacological effects [5]. The bark of *C. cassia* with its characteristic cinnamon aroma is also popular in the food, perfumery, cosmetics and flavor industries [2]; therefore, *C. cassia* is an economically important food and pharmaceutical plant around the world.

It is known that some of the volatile organic compounds (VOCs) contributing to the pleasant aroma have the properties of lipophilia, low molecular weight and high melting points. Studies to date have mainly focused on the VOCs from *C. cassia* barks such as cinnamic acid, cinnamaldehyde, coumarine, flavonoids, and terpenoids [6, 7]. Different cultivars possess different patterns of yield and composition of VOCs [8]. The bark of *C. cassia* is an excellent source of VOCs, and contains 1–2% (v/w) volatile oils, more than 85% (v/v) of which is cinnamaldehyde [3, 8]. Cinnamaldehyde is considered to be the indicator component stipulated in the Chinese pharmacopoeia and the main bioactive component of cinnamon with antifungal, antiparasitic, antitumor, antibacterial, and antidiabetic pharmacological activities [9–11]. Due to differences in cinnamaldehyde content, the variety of *C. cassia*, *Cinnamomum cassia* Presl var. macrophyllum Chu, tastes sweeter, and has a more delicate flavor than *C. cassia* [3]. Relative to cinnamaldehyde, terpenoids have the next largest number of VOCs in *C. cassia* [5]. Terpenoids have also been shown to be vital for the characteristic aroma of plants such as *Wurfbainia longiligularis* [12], *Lonicera japonica* [13], and *Rosa roxburghii* [14]; however, research on the aroma quality of VOCs in *C. cassia* has not been available. Thus, the characterization of aromatic VOCs that influence flavor and aroma differences in *C. cassia* is necessary.

The phenylpropanoid biosynthesis pathway is one of the most important secondary metabolic pathways, associated with the production of cinnamaldehyde, coumarins, flavonoids, lignans, and hydroxycinnamates. This pathway contains several key enzymes such as phenylalanine ammonia-lyase (PAL), cinnamate 4-hydroxylase (C4H), 4-coumarate-CoA ligase (4CL), and cinnamoyl-CoA reductase (CCR) [15]. Several genes encoding key enzymes in the phenylpropanoid pathway, including *PAL*, *4CL*, *CCR*, and beta-glucosidase (*bglB*) were upregulated in *C. cassia* Presl var. macrophyllum Chu relative to *C. cassia* [3]. Key candidate genes associated with the biosynthesis of phenylpropanoids and flavonoids in *C. cassia* were identified from three tissue types: bark, leaves, and branches [16]. The mevalonic acid (MVA) and methylerythritol phosphate (MEP) pathways are two alternative, compartmentally separated pathways in terpenoid biosynthesis [17, 18]. The MEP pathway mainly produces mono- and diterpenes, whereas the MVA pathway is associated with the formation of sesquiterpenes, triterpenes, and sterols [19]. In the MEP pathway, geranyl pyrophosphate (GPP) as the precursor of mono- and di-terpenes is formed from a molecule of dimethylallyl diphosphate (DMAPP) and a molecule of isopentenyl pyrophosphate (IPP) by GPP synthase (GPS) in plastids. Similarly, two molecules of IPP and one molecule of DMAPP could produce the precursor of sesquiterpenes, farnesyl diphosphate (FPP) catalyzed by FPP synthase (FPS) in the cytosol. The terpene synthases (TPSs) also play important roles in catalyzing various reactions on GPP, FPP, and geranylgeranyl diphosphate (GGPP) to generate the C5 carbon skeletons of terpenes and then produce volatile plant terpenoids [20, 21]. Due to the wide application of volatile terpenoids, the aroma profiles of scented plants such as carrot (*Daucus carota* L.) [22], sweet pea (*Lathyrus odoratus*) [23], and *Chrysanthemum indicum* L [24]. have become the focus of more and more attention by researchers. However, the genes responsible for phenylpropanoid and terpenoid biosynthesis remain largely unexplored in *C. cassia*.

In the current study, head-space, solid-phase micro-extraction (HS-SPME) coupled with gas chromatography-mass spectrometry (GC–MS) was employed to analyze the composition and relative content of the VOCs of *C. cassia* bark from five different harvesting times. Transcriptomic and metabolomic analyses were also used to elucidate the regulatory mechanisms of aromatic volatiles by constructing regulatory networks; the key candidate genes were validated using quantitative real-time PCR (qRT-PCR). These results will be helpful in identifying the characteristic aroma of *C. cassia* barks and providing insights into the regularity of aromatic VOC formation in *C. cassia*, which will help to improve the quality of the *C. cassia* medicinal material through biotechnological engineering or marker-assisted breeding methods.

## Results

### Determination of oil cell distribution and specific growth indices

**Fig. 1:**
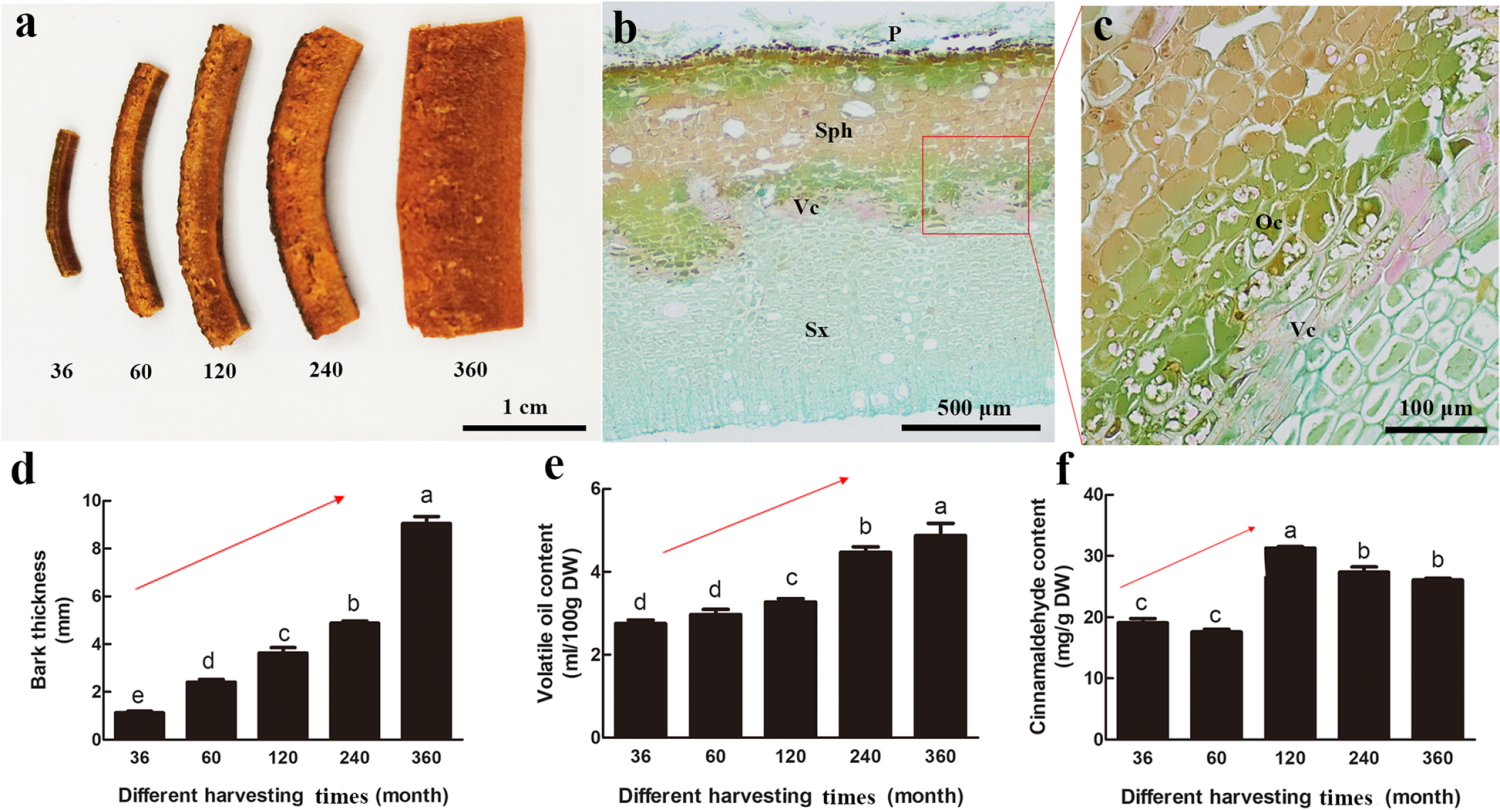
Morphological features and growth indexes of *C. cassia* bark at different harvesting times. **a** Bark morphology. **b** Transverse section of bark at 36 MAP. **c** Oil cells in secondary phloem parenchyma. **d** Bark thickness. **e** Essential oil content. **f** Cinnamaldehyde content. Oc, oil cell; P, periderm; Sph, secondary phloem; Sx, secondary xylem; Vc, vascular cambia

There were distinct phenotypic differences in bark from the five different harvesting times (Fig. 1a). Morphological results showed that oil cells were mainly distributed in the phloem parenchyma near the vascular cambium (Fig. 1b, c). The bark thickness increased significantly with the age and growth of the trees (Fig. 1d), while the volatile oil content increased slowly at the initial period and then increased significantly from 120 MAP to 360 MAP (Fig. 1e). Notably, the cinnamaldehyde content increased markedly from 60 MAP to 120 MAP and then gradually declined (Fig. 1f).

### Volatile metabolome analysis and overall metabolite identification

**Fig. 2:**
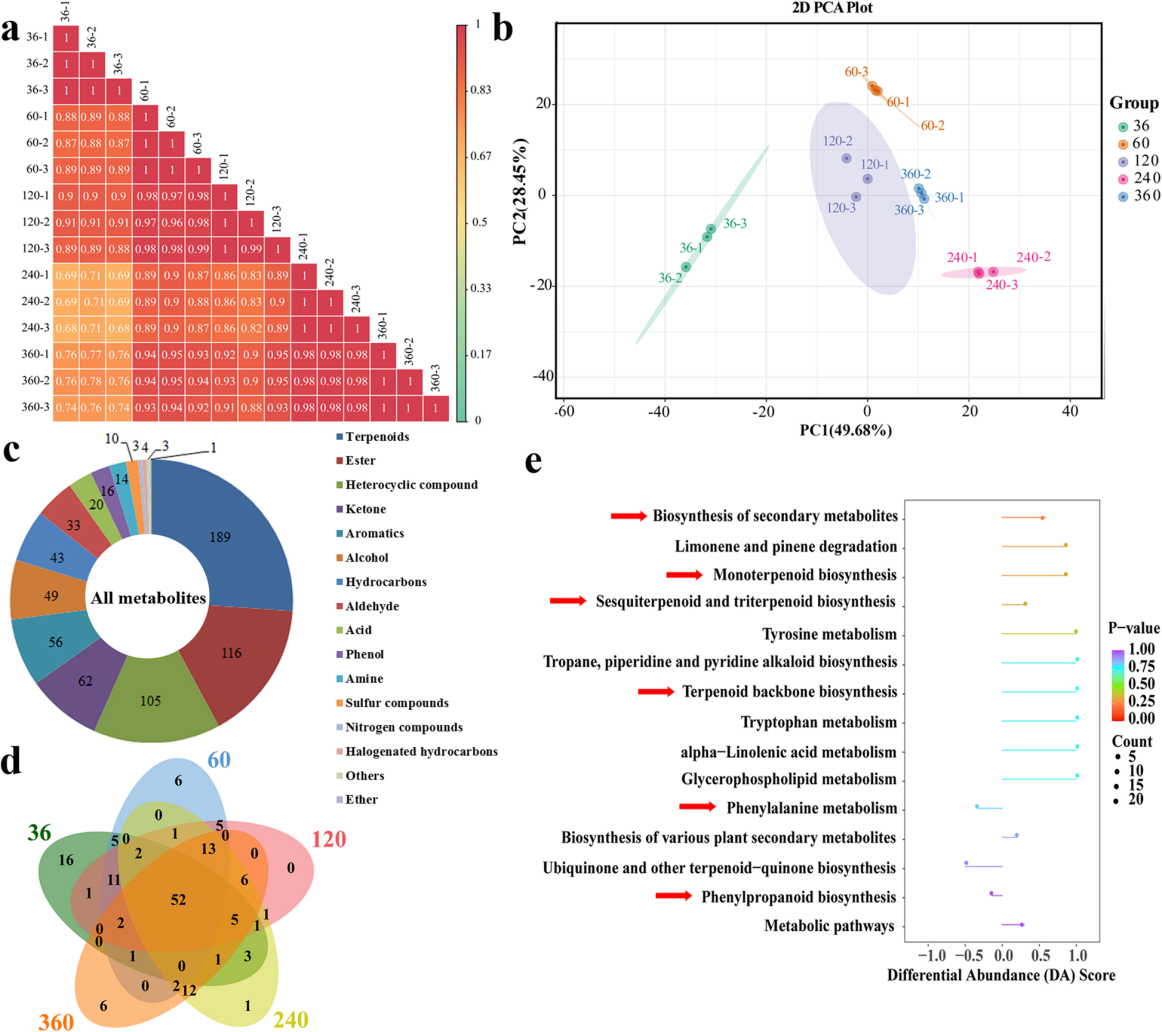
Characterization of differentially accumulated volatiles (DAVs) at different harvesting times. **a** Pearson’s correlation analysis of VOC profiles. **b** Principal component analysis (PCA) of VOC profiles. **c** Component analysis of the identified VOCs. **d** Venn diagram of DAVs by pair-wise comparisons. **e** KEGG enrichment analysis of the DAVs in 60 vs. 240 pairwise comparison. The length of the line segment represents the absolute value of the DA score, and the size of the dot at the endpoint of the line segment represents the number of DAVs involved in the pathway. For dots shown on the left and right panel, the longer the line segment, the more inclined the overall expression of the pathway to be downregulated and upregulated respectively. The color of the line segment and the dot reflects the *p* value

HS-SPME coupled with GC-MS was used to identify and quantitate the VOCs in *C. cassia* bark from different harvesting times, to better understand what contributed to the differences. Correlation among the five different samples was low, but correlation within the replicate samples was high (Fig. 2a). PCA results showed that samples from different harvesting times were dissimilar, but the replicates from the same period were grouped together (Fig. 2b). A total of 748 VOCs were detected, of which 724 were differentially accumulated volatiles (DAVs) (Supplementary Table 2). The DAVs included 189 terpenoids (26%), 116 esters (16%), 105 heterocyclic compounds (15%), 62 ketones (9%), 56 aromatics (8%), 49 alcohols (7%), 43 hydrocarbons (6%), 33 aldehydes (5%), 20 acids (3%), 16 phenols (2%), 14 amines (2%), and 21 others. Among these, terpenoids, esters and heterocyclic compounds accounted for more than half of the total DAVs (Fig. 2c).

All the DAVs were found at each of the five different times, but the concentration varied with the age of the tree. According to the ion intensity, the top100 VOCs at each harvesting period were selected for Venn analysis. We found that there were fifty-two same DAVs among five different harvesting times (Fig. 2d), in which twenty-eight had spicy, woody, sweet, floral, minty and other aroma qualities. Most noteworthy, the high content of cinnamaldehyde with its strong cinnamon scent dominated the aroma quality. Five aromatic terpenoids were also abundant, including α-copaene with a woody, spicy, honey scent, β-bourbonene with an herbal, woody, floral, balsamic scent, α-cubebene with an herbal waxy scent, α-funebrene with a woody odor, and δ-cadinene with a dry, woody scent, suggesting that their scents substantially contribute to the strong aroma of cinnamon bark (Supplementary Table 3).

The number of down-regulated DAVs was higher than the number of up-regulated ones in 36 of 60 pairwise comparisons, whereas the opposite was found in the remaining three pairwise comparisons (Supplementary Fig. S1a). The normalized values of most DAVs with higher levels appeared at 36 MAP and 240 MAP, but most DAVs with lower levels appeared at 60 MAP (Supplementary Fig. S1b). A total of 238 DAVs appeared in four pairwise comparisons (Supplementary Fig. S2). Clustering analysis of the DAVs showed that they could be divided into ten subclasses, four of which peaked at 240 MAP (Supplementary Fig. S3). In 36 vs. 240 pairwise comparisons, the DAVs were enriched in several secondary metabolites, predominantly those related to the biosynthesis of terpenoids such as monoterpenoids, sesquiterpenoids and triterpenoids, as well as phenylpropanoids. In contrast, the DAVs involved in phenylalanine metabolism, ubiquinone and other terpenoid-quinone biosyntheses, were decreased (Fig. 2e).

### Transcriptome analysis of *C. cassiabarks* at different harvesting times

**Fig. 3:**
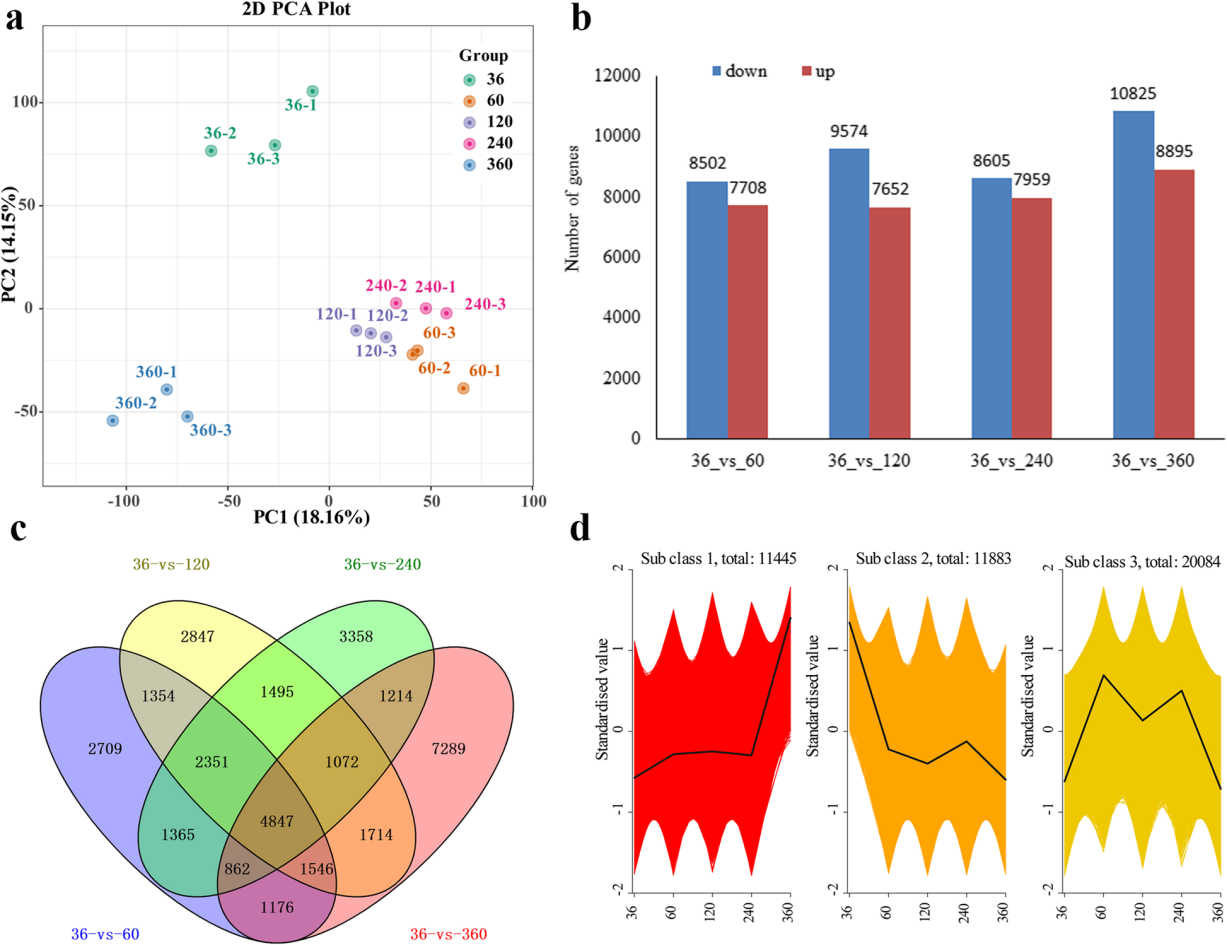
Characterization of differentially expressed genes (DEGs) at different harvesting times. **a** PCA analysis of transcriptome data. **b** Numbers of DEGs in pairwise comparisons. **c** Venn diagrams of DEGs among four comparisons. **d** K-means clustering of DEGs

Transcriptome analysis showed that 105.03 Gb of clean data were obtained (Supplementary Table 4) and a total of 43,412 DEGs were expressed in the five harvesting times (Supplementary Table 5). PCA showed that the transcriptome data from a single period were reproducible, but differed among the times, which indicated that the transcriptome data were satisfactory for further analyses (Fig. 3a). There were differences among the four pairwise comparisons for DEGs (|log_2_fold change|≥1, *p* < 0.05); the minimum DEGs (16,210) were in the 36 vs. 60 stage, with 7708 upregulated and 8502 downregulated genes, while the maximum number (19,720) were in the 36 vs. 360 period, with 8895 upregulated and 10,825 downregulated genes (Fig. 3b). The Venn diagram of DEGs by pairwise comparisons showed 4847 DEGs expressed in common in the four pairwise comparisons (Fig. 3c). A K-means clustering analysis was also performed and the DEGs were mainly divided into three clusters, with 11,445 up-regulated genes (Fig. 3d). KEGG enrichment analysis showed that the top 20 predominantly enriched KEGG pathways were associated with the four pairwise comparisons (Supplementary Fig. S4), and monoterpenoid biosynthesis was highly enriched. In addition, other pathways involved in VOCs biosynthesis were also enriched, including the biosynthesis of phenylpropanoids, flavonoids, and secondary metabolites.

### Construction of the VOCs biosynthesis regulatory network

**Fig. 4:**
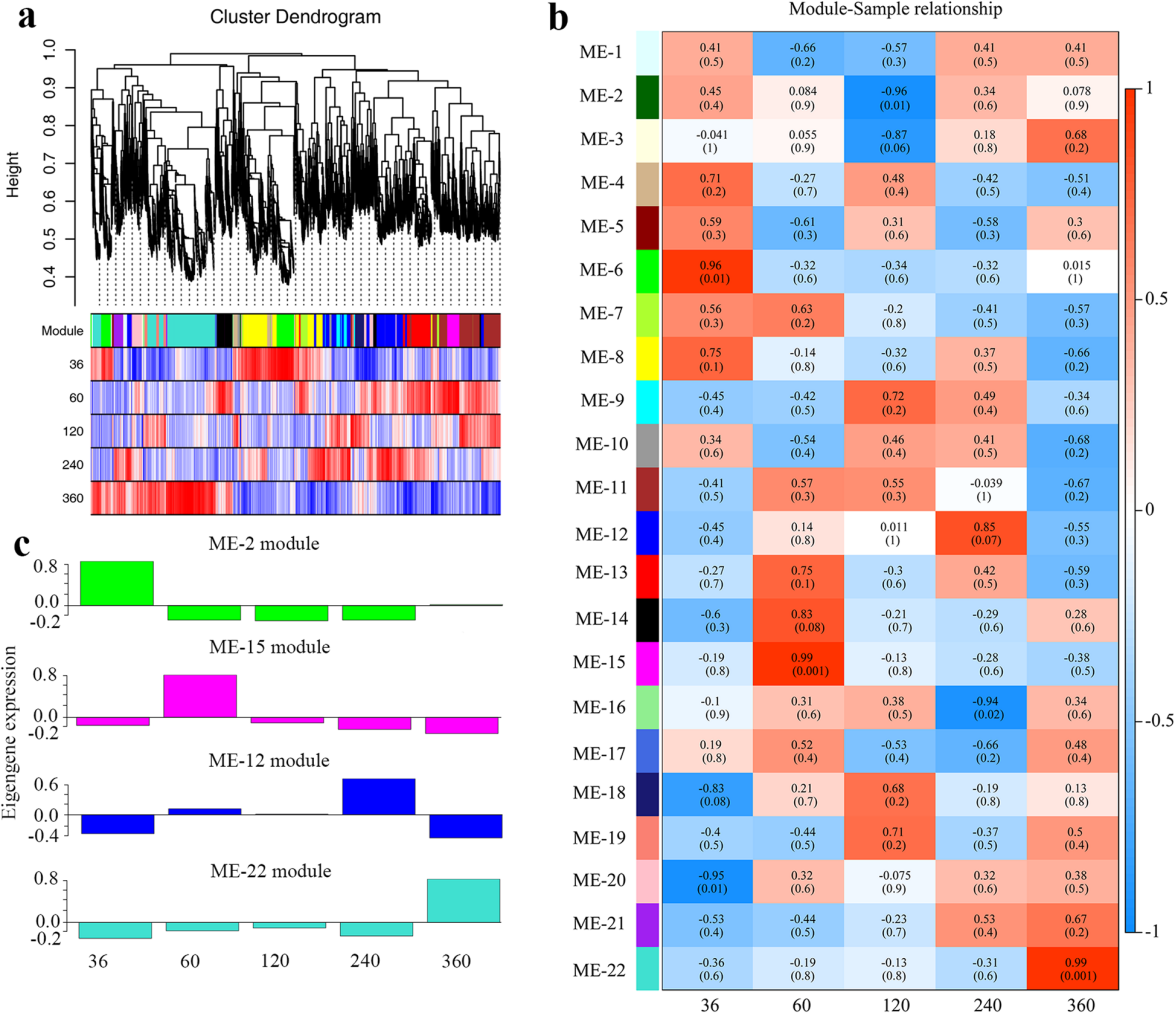
WGCNA analysis in *C. cassia* at different harvesting times. **a** Clustering dendrogram of 11,128 DEGs. **b** Correlations between each module and different harvesting times. Pearson correlation coefficients and *p* values (in brackets) are indicated in the grid where modules and traits intersect. **c** Eigengene expression of four modules with strongly positive correlations

To determine the regulatory network of VOC biosynthesis in bark, weighted co-expression network analysis (WGCNA) was conducted on the five different harvesting times to identify the modules with high gene co-expression. A total of 11,128 genes was screened (Fig. 4a), and 22 modules with similar expression patterns were identified by hierarchical clustering (Fig. 4b). Four co-expression modules had highly positive correlations with harvesting times (*r* > 0.85), including ME-6 at 36 MAP, ME-15 at 60 MAP, ME-12 at 240 MAP, and ME-22 at 360 MAP (Fig. 4c), suggesting that the genes in these modules might be involved in the processes of VOC biosynthesis.

A total of 123 transcription factors (TFs) were differentially expressed in the four co-expression modules (2, 12, 15, and 22), and were classified into 21 TF families. Among these, almost half of the TFs were distributed among six families: basic helix–loop–helix (bHLH), WRKY, C2H2, B3-ARF, TCP, and GRAS. Several TFs, such as bHLH23, C2H27, and WRKY1, were significantly up-regulated from 60 MAP to 360 MAP (Supplementary Table 6).

### Mechanism of VOC accumulation in the phenylpropanoid biosynthesis pathway

**Fig. 5:**
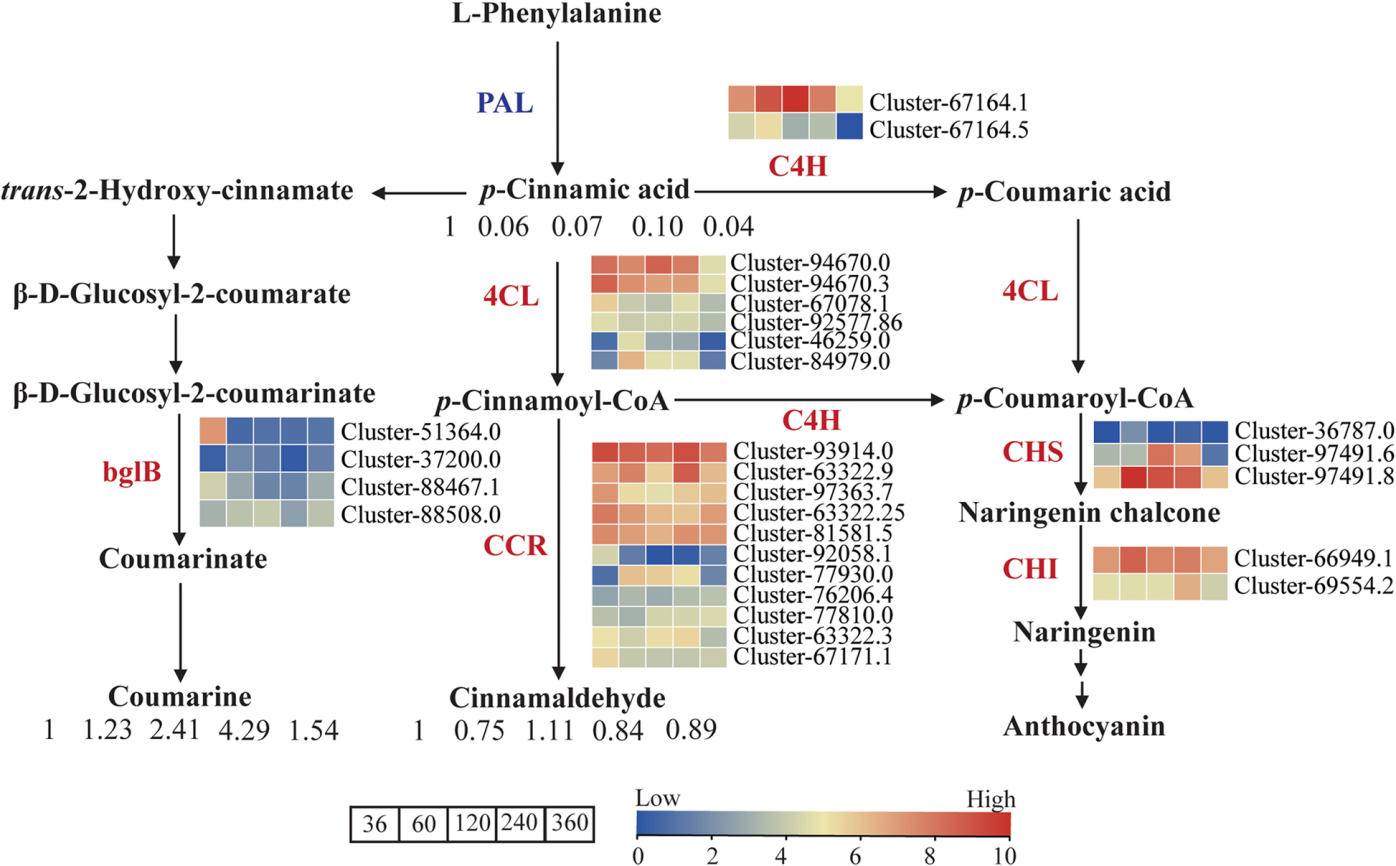
Phenylpropanoid biosynthesis pathway in *C. cassia* at different harvesting times. The pathway is simplified from Gao et al. [3] and obtained the copyright permission by Kanehisa laboratories. Log10 (FPKM) was calculated to represent the expression levels of each gene. The redder colors indicate higher expression levels. The numbers under the volatiles represent the fold change compared to 36 MGP according to metabolite ion intensity. The grids within a row and the numbers under the volatiles from left to right correspond to the 36, 60, 120, 240, and 360 MGP. The DEGs are marked in red

Three DAVs in the phenylpropanoid biosynthesis pathway, *p*-cinnamic acid, coumarine, and cinnamaldehyde, were identified by metabolomics analysis (Fig. 5); the ion intensity of cinnamaldehyde was much higher than the others (Supplementary Table 2). The metabolite ion intensity of cinnamaldehyde was highest at 120 MAP, while *p*-cinnamic acid and coumarine were highest at 36 MAP and 240 MAP, respectively.

Transcriptomics analysis revealed 28 DEGs involved in phenylpropanoid biosynthesis in the four co-expression modules: *4CL*, *C4H*, *bglB*, *CHS*, *CHI*, and *CCR* (Supplementary Table 7). Most of these were up-regulated in the process of rapid VOC accumulation. In addition, the genes encoding key enzymes involved in cinnamaldehyde biosynthesis have multiple homologous genes. For example, *CCR* has 11 homologous genes, most of which were up-regulated at 36 or 240 MAP, especially cluster-93914.0, cluster-63322.9 and cluster-81581.5 (Fig. 5). Six genes were predicted to encode 4CL, and the expression profiles of cluster-94670.0 and cluster-94670.3 were significantly positively correlated with the accumulation of cinnamaldehyde (Fig. 1f).

### Mechanism of VOC accumulation in the terpenoid biosynthetic pathway

**Fig. 6:**
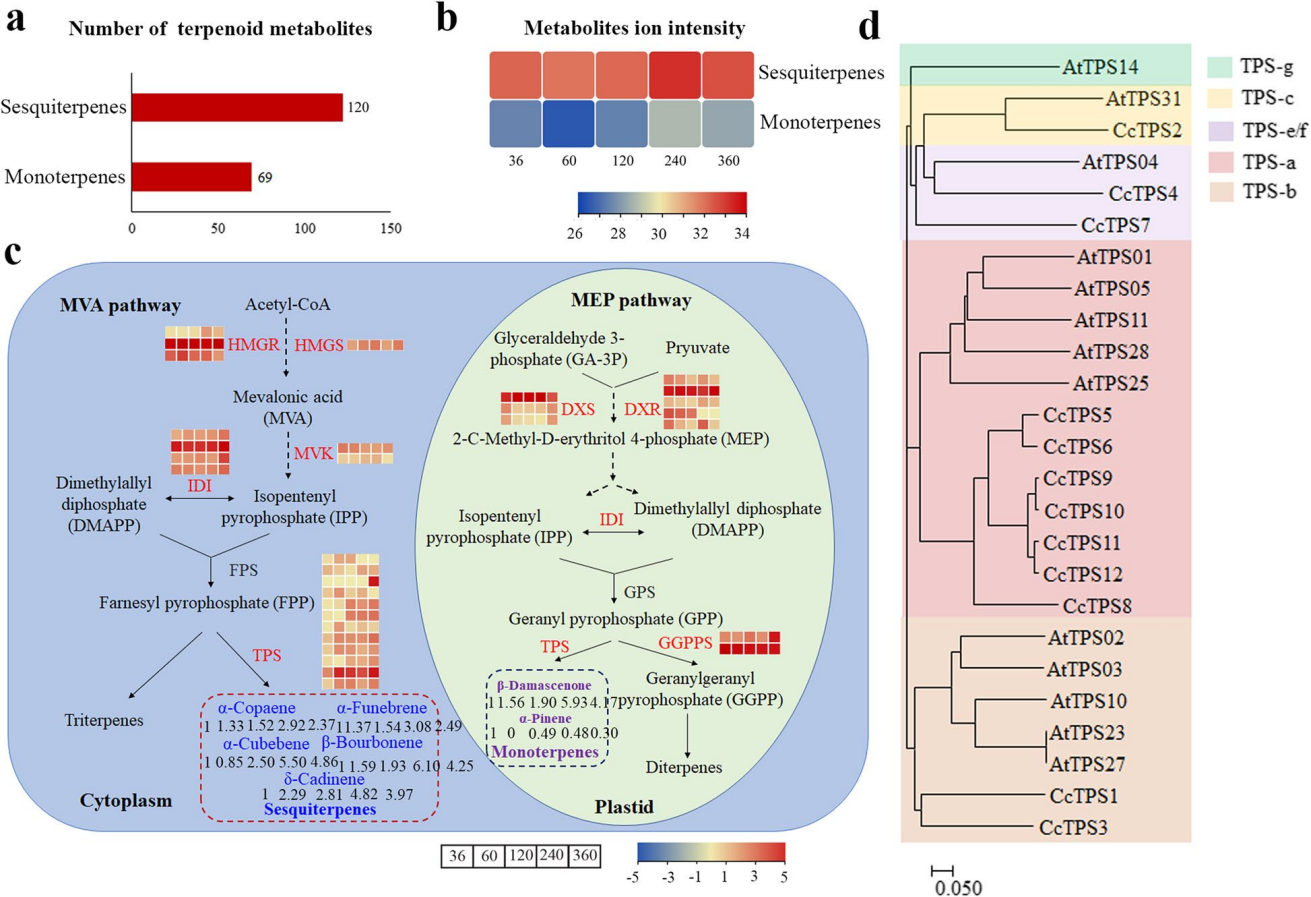
Terpenoid biosynthesis pathway in *C. cassia* at different harvesting times. **a** Number of terpenoid metabolites. **b** Metabolite ion intensities between monoterpenes and sesquiterpenes. **c** Terpenoid biosynthesis pathway. The pathway is simplified from Li et al. [13] and copyright permission was obtained from Kanehisa Laboratories. The expression levels of each gene are represented by log10 (FPKM). The redder colors indicate higher expression levels. The numbers under the volatiles represent the fold change compared to 36 MGP according to metabolite ion intensity. The grids within a row and the numbers under the volatiles from left to right correspond to 36, 60, 120, 240, and 360 MGP. The DEGs are marked in red. **d** Phylogenetic tree of CcTPS genes

In this study, the terpenoid DAVs consisted of 120 sesquiterpenes and 69 monoterpenes, and the number of sesquiterpenes was 1.72-fold greater than that of monoterpenes (Fig. 6a). The metabolite ion intensity of sesquiterpenes and monoterpenes was also highest at 240 MAP, followed by 360 MAP (Fig. 6b). Among them, the top DAVs were all sesquiterpenes according to the ranking of their metabolite ion intensity, including α-copaene, α-funebrene, α-cubebene, β-bourbonene and δ-cadinene, which were significantly accumulated at 240 MAP. The monoterpenes, β-damascenone and α-pinene, were highest at 36 and 240 MAP, respectively (Fig. 6c and Supplementary Table 2).

In the MEP pathway, 3 *DXSs*, 5 *DXRs*, and 2 *GGPPSs* were identified from the four co-expression modules, and most were up-regulated during monoterpene accumulation. Most of the genes in the MVA pathway, including *HMGRs*, *HMGSs*, *IDIs*, *MVKs* and *TPSs*, were up-regulated at 240 MAP. Twelve TPSs were identified, and nine were up-regulated in the late stage of bark development (Fig. 6c and Supplementary Table 7). According to the phylogenetic tree constructed using TPSs from *Arabidopsis thaliana* (Supplementary Table 8) and *C. cassia*, seven TPSs, including TPS5, TPS6, TPS8, TPS9, TPS10, TPS11, and TPS12, were divided into the TPS-*a* subfamilies. TPS4 and TPS7 were assigned to the TPS-*e/f* group, whereas TPS1 and TPS3 were distantly related to the TPS-*b* group. TPS2 clustered together with AtTPS31, which belonged to the TPS-*c* group. All the TPSs from the TPS-*a* sub-families were up-regulated along with the bark harvesting process. TPS3 had an especially high expression level at 360 MAP (Fig. 6d).

### Gene regulatory network associated with aromatic VOC metabolites

**Fig. 7:**
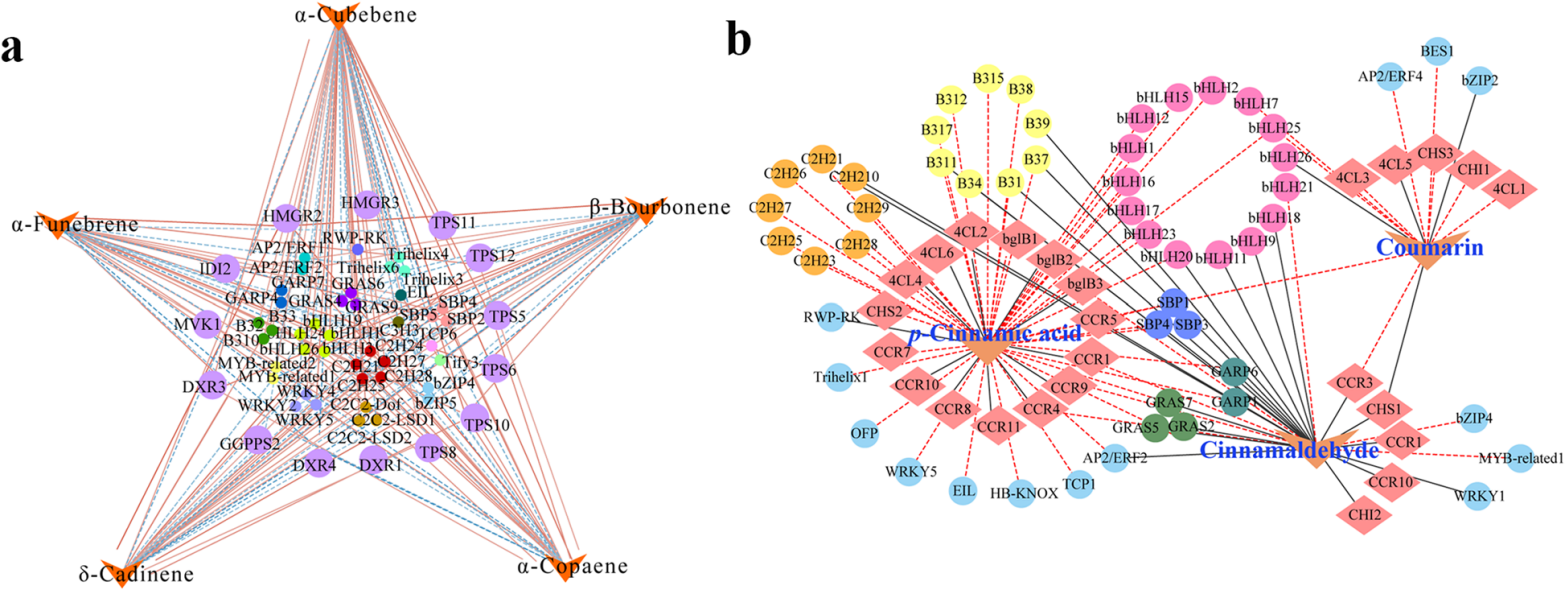
Regulatory network for aromatic VOC biosynthesis in *C. cassia*. **a** Five characteristic sesquiterpenes. The solid lines in red represent positive correlations. **b** Three aromatic VOCs in phenylpropanoid biosynthesis pathway. The lines in black represent positive correlations

In light of these findings, a gene-metabolite regulatory network of aromatic terpenoids and phenylpropanoid metabolism was constructed using Pearson’s correlation coefficients (Fig. 7). For aromatic terpenoid metabolism, a total of 123 TFs and 32 DEGs involved in terpenoid synthesis were identified from the four co-expression modules. Among them, 41 TFs were predicted to regulate fourteen structural genes to synthesize five sesquiterpenoids, α-copaene, α-funebrene, α-cubebene, β-bourbonene and δ-cadinene. The TFs included five bHLHs, five C2Hs, three WRKYs,three SBPs, three C2C2s, three Trihelixs, three GRASs, three B3-ARFs, two AP2/ERFs, two MYB-relateds, two GARPs, two bZIPs, one Tify, one EIL, one RWP-RK, one C3H, and one TCP. The structural genes in the regulatory network of aromatic terpenoid metabolism were six *TPSs*, two *HMGRs*, three *DXRs*, *IDI2*, *MVK1*, and *GGPPS2*. Four TPSs, TPS5, TPS6, TPS10, and TPS12, might be activators in regulating the biosynthesis of these five aromatic sesquiterpenoids, while TPS11 might play a negative role in regulating aromatic sesquiterpenoid biosynthesis. The TFs, bHLH1 and bHLH3, were predicted to positively regulate aromatic sesquiterpenoid biosynthesis, but bHLH19 might play an opposite role. AP2/ERF2 and WRKY5 might also positively regulate the biosynthesis of these five aromatic sesquiterpenoids (Fig. 7a).

For aromatic phenylpropanoid metabolism, *p*-cinnamic acid, coumarine and cinnamaldehyde were highly correlated with fifteen, five and five structural genes, respectively. Notably, *p*-cinnamic acid was positively regulated by ten structural genes, including three *4CLs*, two *bglBs*, and five *CCRs*. Cinnamaldehyde was the main aromatic phenylpropanoid, and its biosynthesis was positively regulated by five structural genes, primarily *CCR1*, *CCR3*, and *CCR10*. These three VOCs were highly correlated with fifty-five TFs in the phenylpropanoid metabolic network. The TFs, bHLH9, bHLH11, bHLH18, bHLH20, and bHLH21, were predicted to be activators in regulating cinnamaldehyde biosynthesis, whereas all the bHLHs could negatively regulate the biosynthesis of *p*-cinnamic acid. Similar to the bHLHs, all the B3-ARFs could negatively regulate the biosynthesis of *p*-cinnamic acid. The B3-ARFs, B31, B34, B37, B39, and B311, were predicted to positively regulate the biosynthesis of cinnamaldehyde (Fig. 7b).

### Verification of key candidate genes related to VOC biosynthesis

**Fig. 8:**
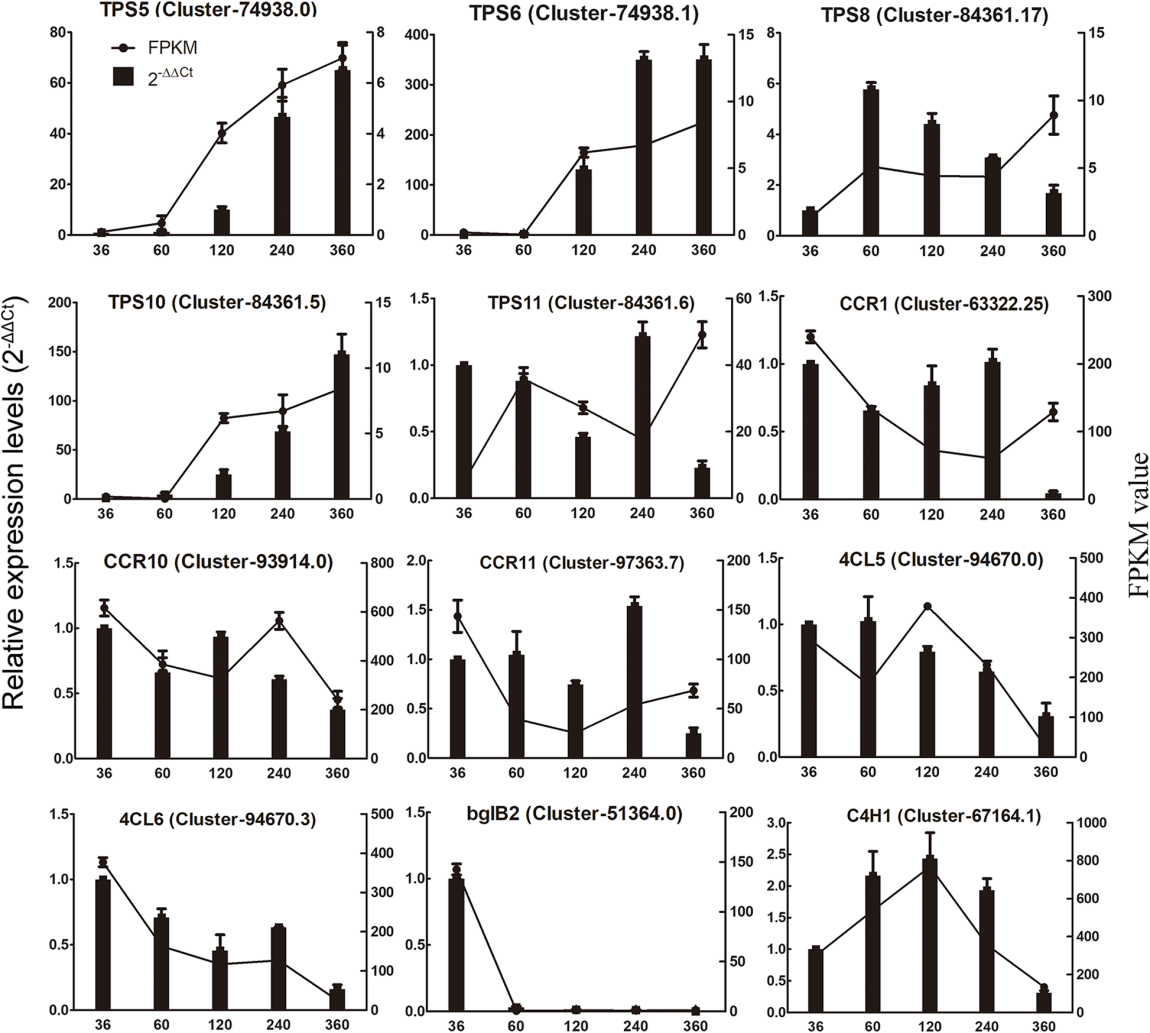
Expression profiles of candidate genes involved in the phenylpropanoid and terpenoid biosynthetic pathways

The expression patterns of twelve key candidate genes involved in the biosynthesis of terpenoids and phenylpropanoids were determined by qRT-PCR (Fig. 8). The results showed that the trends of FPKM values from transcriptome data were consistent with the relative gene expression levels (2^−ΔΔCt^), indicating that the transcriptome data were reliable and accurate. Furthermore, the candidate genes selected from regulatory networks were further confirmed by their relative expression levels using qRT-PCR. These key genes included *CCRs*, *4CLs*, *bglB2*, *C4H1*, involved in the phenylpropanoid biosynthetic pathway, and *TPS5*, *TPS6*, *TPS8*, *TPS10*, and *TPS11*) in the terpenoid biosynthetic pathway.

## Discussion

Previous studies have demonstrated that almost all *Cinnamomum* species including *Cinnamomum osmophloeum* [25], *Cinnamomum camphora* [26], and *C. cassia* [27] contain VOCs, including terpenes, terpenoids, and phenylpropanoids. Aldehydes, lipids and terpenoids were shown by in situ histochemical assay to be localized to *C. cassia* oil cells, where aldehydes were distributed in the area surrounding the oil sac [2]. Our results also showed the concentration of oil cells mainly in the phloem parenchyma near the vascular cambium of bark (Fig. 1b, c). The composition of VOCs in different tissues [16, 28] and varieties [3] significantly varies in *C. cassia*, so the variety with the desired aroma should be selected to meet consumer preferences. However, no study has determined the differences in VOCs in *C. cassia* at different harvesting times. In this study, 724 differentially accumulated VOCs were identified, including terpenoids (26%) and aldehyde (5%) (Fig. 2c). A great deal of essential oil profiles and chemotypes have been identified from *Cinnamomum* species, in which terpenoids and phenylpropanoids occupied more than 50% and were regarded as the primary components [29]. Cinnamaldehyde was proven to be the most plentiful metabolite in the essential oil of *C. cassia* [30] and *C. zeylanicum* [31]. Sandner et al. identified 24 terpenoids from buds of *C. cassia*, but cinnamaldehyde (40 mg/g) was the most abundant compound [28]. We identified twenty-eight aromatic VOCs from buds of *C. cassia*, and cinnamaldehyde was the most abundant, peaking at 120 MAP with its spicy cinnamon odor dominating the aroma qualities (Fig. 1). In addition, many studies have found that floral scents and fruit flavor were mainly attributed to terpenoids [13, 23, 32]. The terpenoids, α-copaene, β-bourbonene, α-cubebene, α-funebrene, and δ-cadinene, were also considered aromatic VOCs that contributed to the cinnamon odor of *C. cassia*, through their strong herbal and woody aromas (Supplementary Table 3).

In recent years, the integration of transcriptomics and metabolomics in medicinal plant research has facilitated elucidation of the biosynthetic mechanisms and associated major metabolic pathways [33]. The transcriptome analysis in this study revealed that 43,412 DEGs were significantly enriched in the terpenoid, phenylpropanoid, and flavonoid biosynthetic pathways (Supplementary Fig. S4), which coincided with the metabolomics results. The biosynthesis of *p*-cinnamic acid, cinnamaldehyde and coumarine are synergistically regulated by structural genes involved in the phenylpropanoid biosynthesis pathway [3]. The compound, *p*-cinnamic acid, was enzymatically converted to *p*-cinnamoyl-CoA by 4CL, and then modified by CCR to yield cinnamaldehyde [34]. Consistent with previous studies [3, 16], *p*-cinnamic acid and cinnamaldehyde accumulated differentially at different bark harvesting times (Fig. 5), and could be positively regulated by *4CL2*, *4CL4*, and *4CL6*, and *CCR1*, *CCR3*, and *CCR10*, respectively (Fig. 7). Terpenoids are deemed to be the most abundant secondary metabolites in plants due to their diverse structures and aromatic scents [35]. In this study, 189 mono- and sesqui- terpenes, which were the most abundant metabolites in VOCs, were identified in *C. cassia* bark (Fig. 6a). In the terpenoid metabolic pathways, numerous terpenes are synthesized under the spatial and temporal control of several key enzymes by undergoing multiple reactions [36]. Previous studies revealed that the expression profiles of genes encoding key enzymes such as GGPPS, DXS, MVK, and TPS, were positively correlated with terpenoid yield, which is crucial for high-quality flavor [13, 37, 38]. Consistent with these earlier findings, the higher expression of several upstream genes (*DXRs*, *HMGRs*, *HMGSs*, *IDIs*, *MVK1* and *GGPPSs*) in the rapid-accumulation stages (240 and 360 MAP) of *C. cassia* might enhance the metabolic flux toward the precursors of terpenoid biosynthesis (Fig. 6c).

TPSs play important roles in controlling the biosynthesis of the diverse terpenoids in plants [39]. It has been proven that the tremendous diversity of volatile terpenoids is mainly attributable to the catalytic versatility of TPSs, many of which use only GPP, FPP, and GGPP as substrates to generate multiple volatile products from prenyl diphosphate precursors [18]. In recent decades, an increasing number of individual volatile TPS genes have been identified and characterized in plants [23, 40]. However, the TPS genes of *C. cassia* in the terpenoid biosynthetic pathways remain unknown. In the current study, twelve TPSs were identified in *C. cassia*, nine of which were up-regulated during the rapid accumulation times of VOCs (Fig. 6c). TPS in plants that yield terpenoids usually have a common phylogenetic origin and have been divided into seven subfamilies (TPS-*a* to TPS-*g*) [41]. Because the TPS-*f* subfamily split off from the base of the TPS-*e* subfamily, it is usually designated as the TPS-*e*/*f* subfamily. AtTPS04 was classified to the TPS-*e*/*f* subfamily, which has been reported to produce the diterpene, geranyllinalool in *Arabidopsis* [42]. TPS4 and TPS7 in *C. cassia* are clustered together with AtTPS04 (Fig. 6d), indicating that they might be involved in regulating the diterpene biosynthetic pathway. In gymnosperms, all the TPSs were classified to the gymnosperm-specific subfamily, TPS-*d*, and most of the characterized TPSs in the TPS-*a* clade are sesquiterpene synthases [41]. In this study, seven TPSs (TPS5, TPS6, TPS8, TPS9, TPS10, TPS11, and TPS12) were assigned to the TPS-*a* subfamily (Fig. 6d). Among them, five TPSs (TPS5, TPS6, TPS8, TPS10, and TPS12) and TPS11might be involved in regulating the biosynthesis of sesquiterpenoids by a gene-metabolite regulatory network–especially the main aromatic sesquiterpenoids. The TPSs in the TPS-*b* subfamily were reported to function specifically in flowering plants to synthesize cyclic monoterpenes or isoprenes [41]. Phylogenetic results in our work showed that two genes homologous to AtTPS03, TPS1 and TPS3, belonged to the TPS-*b* group, together with AtTPS03, AtTPS10, AtTPS23 and AtTPS27, which have been reported to regulate the synthesis of the monoterpene (*E*)-β-ocimene [43], α-farnesene [41], β-myrcene and 1,8-cineole [44] in *A. thaliana*. Surprisingly, the functional redundancy and convergence of TPSs were also observed in plants. For example, three TPSs controlling nerolidol synthesis were distinct in TPS subfamily lineages, while two TPSs from different TPS subfamilies controlled linalool synthesis in red_5_ kiwifruit (*Actinidia spp.*) [40]. Thus, although the identification of key candidate TPSs, which might be associated with volatile terpenoid biosynthesis, were identified in this study, the comprehensive TPS gene identification, expression and functional analysis needs further elucidation.

Many TFs have been confirmed to be associated with the regulation of secondary metabolism of plants by binding to the promoters of structural genes to activate the co-expression of multiple genes in a metabolic pathway. To date, the TFs AP2/ERF, MYB, C2H2, bHLH, bZIP, and WRKY have been studied extensively, and found to regulate the production of aromatic VOCs in various plant species [13, 45, 46]. In *C. cassia*, AP2/ ERF, MYB, C2H2, bHLH, and WRKY were significantly differentially expressed in bark, branches, and leaves [16] and in different varieties (*C. cassia* and *C. cassia* var. macrophyllum Chu) [3]. The present study determined the transcriptome profile of bark at five successive harvesting times, and demonstrated that certain TF family members such as bHLH, AP2/ERF, WRKY, B3-ARF, MYB, C2H2, GRAS, and bZIP were significantly differentially expressed and predicted to be involved in regulatory networks of the main aromatic VOCs in *C. cassia* (Fig. 7). The functions of many bHLH proteins in plants have been studied in detail. For example, PpbHLH1, a homologous gene to bHLH1in *C. cassia*, was activated by PpTPS3 via direct binding to the promoter to positively enhance flavor-related linalool production and thus improve the flavor of the fruit in *Prunus persica* [47]. In *Artemisia annua*, AabHLH1 positively regulated artemisinin biosynthesis by binding to the E-box cis-elements in both ADS and CYP71AV1 promoters [48]. The MYB-bHLH‐WD40 (MBW) complex usually plays an important role in regulating flavonoid biosynthesis. The strawberry FaEGL3 (bHLH, GeneBank: MW700313) interacted with R2R3‐FaMYB5 and FaLWD1/FaLWD1‐like to form an MBW complex, positively regulating the accumulation of anthocyanins and proanthocyanidins [49]. In this regulatory network, bHLH9, bHLH11, bHLH18, bHLH20, and bHLH21 played possible roles in the regulation of cinnamaldehyde metabolism, while bHLH1, bHLH3, and bHLH19 with the higest homology to MYC2-like, bHLH79-like, and MYC in *Cinnamomum micranthum* f. kanehirae, respectively [50], were predicted to regulate sesquiterpenoid biosynthesis (Fig. 7). However, no data were available from this study on whether bHLH proteins regulated the formation of aromatic VOCs by the MBW complex, which warrants further investigation. WRKY TFs have also been reported to play important roles in positively regulating the biosynthesis of volatile terpenes and phenylpropanoids, such as AaWRKY1 in *Artemisia annua* [51], IiWRKY34 in *Isatis indigotica* [52], and GhWRKY41 in *Gossypium hirsutum* (cotton) [53]. WRKY2, WRKY4, and WRKY5 were significantly upregulated and highly correlated with the biosynthesis of aromatic sesquiterpenoids, and had high homology to IiWRKY34 and AaWRKY1, thus might contribute to the production of these sesquiterpenoids in *C. cassia*. WRKY1, with high homology to GhWRKY41 by regulating phenylpropanoid metabolism [53], might also play an important role in cinnamaldehyde biosynthesis (Fig. 7a and Supplementary Table 6). An AP2/ERF family transcription factor, ZmEREB58, was found to be a positive regulator of sesquiterpene metabolism by directly promoting TPS10 expression in maize [54], while NtERF13a enhanced phenylpropanoid biosynthesis in tobacco [55]. AP2/ERF family transcription factors might play roles in regulating phenylpropanoid and sesquiterpenoid biosynthesis (Fig. 7). In addition, ARFs (auxin response factors) belonging to the plant-specific B3 superfamily, were reported to specifically target the upstream promoter regions of many auxin response genes and to heteromerize with Aux/IAA proteins in the auxin signal transduction pathway active in various processes of plant growth and development [56]. For instance, in *Populus trichocarpa*, the genes PtrARF4, PtrARF18, and PtrARF35 could play important roles in regulating early development processes of wood formation by improving cambium differentiation and xylem expansion [57]. Here, seventeen ARFs were identified during the process of tree growth and development. Among them, B31, B34, B37, B39, and B311 had higher expression levels during cinnamaldehyde synthesis, while B32, B33, and B310 were active in terpenoid biosynthesis (Fig. 7a and Supplementary Table 6), suggesting that these genes might participate in regulating aromatic VOC accumulation via improving cambium differentiation and xylem expansion during tree growth and development. Considering the identification of these differentially regulated TFs at different harvesting times and the homologous genes which function had been reported in other plants, some key candidate TFs might be targeted, which will pave the way for revealing the regulatory mechanism of aromatic VOC metabolism.

## Conclusions

In this study, we found significant quantitative differences in *C. cassia* bark from different harvesting times by phenotypic, cytological, transcriptomic and metabolomic analyses. Aroma, which is mainly determined by VOCs, is an attribute affecting the flavor and medicinal properties of *C. cassia*. The results showed that cinnamaldehyde and terpenoids, especially the sesquiterpenes, α-copaene, β-bourbonene, α-cubebene, α-funebrene, and δ-cadinene, were the main aromatic components of *C. cassia* barks. The greater accumulation of cinnamaldehyde and total VOCs in *C. cassia* bark might be the reason for better aroma quality in processing. A number of DEGs and TFs related to VOC biosynthesis were also identified by integrating transcriptomics and metabolomics analyses; 120 MAP and 240 MAP might be important for forming cinnamaldehyde and terpenoids, respectively. Finally, a gene-metabolite regulatory network was constructed to identify potential candidate genes and TFs that might be involved in terpenoid and phenylpropanoid biosynthesis in *C. cassia*. This paper is the first to systematically research the metabolic pathways and molecular regulation mechanisms of aromatic VOCs in *C. cassia*, which provides a theoretical basis for the molecular breeding of high-quality cinnamon.

## Methods

### Plant materials

The bark from five different harvesting times, 36, 60, 120, 240, and 360 MAP, of *Cinnamomum cassia* Presl was collected in Guiping city, Guangxi, China (23°04′43″ N, 110°25′62″ E). The samples were identified by Zhonghua Dai, associate professor at the Guangxi University of Chinese Medicine. The bark from three individual trees was stripped off and mixed together as one biological replicate, and three biological replicates were collected for each harvesting period. The samples were quickly frozen in liquid nitrogen and stored at − 80℃ until needed.

### Determination of bark thickness, volatile oil and cinnamaldehyde content, and morphology

The thickness of fresh bark was measured using a digital electronic caliper. After drying to constant weight at 45℃, the dry weight (DW) of the bark was measured. The content of cinnamaldehyde and volatile oil was determined by a method from the Pharmacopoeia of the People’s Republic of China [4]. The anatomical and histological examination of the bark followed our previous method [58].

### HS-SPME and GC–MS analysis

Dried bark samples were ground in liquid nitrogen and 500 mg (~ 1 mL) of powder was placed in a 20 mL headspace vial (Agilent, Palo Alto, CA, USA) with 2 mL of saturated NaCl solution and 10 µL of internal standard (50 µg/mL). TFE-silicone headspace septa (Agilent) were used to seal the vials, which were heated at 60 °C for 5 min. For GC–MS analysis, a 120 μm divinylbenzene/carboxen/polydimethylsiloxane fiber was exposed to the headspace of the samples for 15 min at 60 °C.

The composition of the VOCs was determined and specific compounds were quantified using an Agilent model 8,890 GC and a model 7000 MS (Agilent Technologies, Stockport, United Kingdom), equipped with a DB-5MS capillary column (30 m × 0.25 mm × 0.25 μm). The parameters were programmed as follows: linear velocity, 1.2 ml/min; oven temperature, 40 °C for 3.5 min to start, then rising at 10 °C/min, 7 °C/min, and 25 °C/min to 100 °C, 180 °C, and 280 °C, respectively; and held for 5 min at 280 °C. The MS was operated in electron impact (EI) ionization mode at 70 eV. The temperature of the quadrupole mass detector was 150 °C, the ion source was 230 °C, and the transfer line was 280 °C. The composition and quantification of VOCs was determined in ion monitoring (SIM) mode. MassHunter quantitative analyses and internal standard normalization were used to calculate the peak area and relative content of each compound.

Principal component analysis (PCA) and hierarchical cluster analysis (HCA) were performed using the statistics function, Procomp, within the R package and presented as a heatmap. Differentially accumulated volatiles (DAVs) were obtained using a threshold of ≥ 1.0 for the absolute value of log_2_FC (|log2FC|) and a VIP value ≥ 1 for two-group analysis. The annotated DAVs were mapped in the KEGG pathway database and the main KEGG pathways were selected for further analysis. Because all samples contributed to the cinnamon aroma, the 100 most abundant VOCs in each harvesting period were selected for analysis to identify the primary VOCs. The aroma qualities of the commonest VOCs were obtained from the literature and the ChemicalBook online software (https://www.chemicalbook.com/).

### RNA extraction, cDNA library construction, sequencing, and determination of differentially expressed genes (DEGs)

Total RNA was isolated using an RNAprep Pure Plant kit (Tiangen, Beijing, China) according to the instructions. The purity and integrity of the RNA were assessed using a NanoDrop micro-spectrophotometer (Thermo Fisher Scientific, Wilmington, DE, USA) and an Agilent 2,100 Bioanalyzer (Agilent Technologies, Santa Clara, CA, USA), respectively. The cDNA libraries were sequenced by Metware Biotechnology Co., Ltd. (Wuhan, China) on an Illumina sequencing platform. Three biological replicates for each sample were done.

To annotate the gene functions, all the clean reads were mapped to NR, Pfam, NT, KEGG, GO, Swiss-Prot, and KOG/COG databases. The fragments per kilobase per million mapped reads (FPKM) of each gene were calculated and PCA was carried out in R software to determine correlations between repetitions. The DEGs were identified with a threshold of |log_2_fold change|≥ 1 and *p* value < 0.05.

### Correlation analysis of transcriptomic and metabolomic data

A weighted gene correlation network analysis (WGCNA) was conducted using the WGCNA v1.68 software package [59], where the FPKMs of DEGs from 15 samples were used as the input data, to identify the highly correlated expression of gene clusters. The highly correlated genes clustered to the same module, and are represented by different colors on the cluster tree. Next, highly correlated modules were identified to construct the regulatory networks. Transcription factors (TFs) were also identified from the modules with high correlation coefficients. The transcript-metabolite network was constructed with a Pearson’s correlation coefficient threshold of > 0.8 and *p* < 0.05 between DEGs and DAVs.

### Quantitative real-time reverse-transcriptase PCR (qRT-PCR) analysis

Total RNA was extracted from bark samples, and cDNA was synthesized using the PrimeScript RT reagent kit with gDNA Eraser (Takara, Beijing, China). Twelve candidate genes were selected for qRT-PCR analysis. The internal reference gene was glyceraldehyde-3-phosphate dehydrogenase (GAPDH) [60]. The sequencing primers were designed using Primer-5 software (Supplementary Table 1). The qRT-PCR conditions were the same as previously reported [58].

### Statistical analyses

Significance was tested by ANOVA and multiple comparisons were performed using IBM SPSS Statistics 24.0 software. Graphpad prism 5.0 software was used to create figures. Values marked with different lowercase letters are significantly different (*p* < 0.05).

### Supplementary Information

**Additional file 1: Supplementary Fig. S1.** Differentially accumulated volatiles (DAVs) in different harvesting times. **Supplementary Fig. S2.** The venn diagrams of DAVs among four comparisons. **Supplementary Fig. S3.** K-means clustering analysis of the DAVs. **Supplementary Fig. S4.** Significantly enriched KEGG pathways of DEGs.

**Additional file 2: Supplementary Table 1.** Primers of genes used for qRT-PCR analysis. **Supplementary Table 2.** Total identified Differentially accumulated volatiles. **Supplementary Table 3.** The identified metabolites from top-100 abundant VOCs in each harvesting time and their aroma qualities. **Supplementary Table 4.** Summary of the BGISEQ-500 RNA-Seq data analysis. **Supplementary Table 5.** Total identified DEGs. **Supplementary Table 6.** Transcription factors in the four modules (green, blue, magenta, and turquoise). **Supplementary Table 7.** Identification of DEGs involved in phenylpropanoid and terpenoid biosynthesis pathway. **Supplementary Table 8.** TPS proteins from* Arabidopsis thaliana* used in phylogenetic analysis.

## Data Availability

All datasets generated or analyzed during this study are available from the corresponding author upon reasonable request. The raw data was uploaded to Sequence Read Archive (PRJNA1001064) (https://www.ncbi.nlm.nih.gov/search/all/?term=PRJNA1001064).
