# Supplementary material for: Integrated transcriptomics and metabolomics analysis provides insights into aromatic volatiles formation in Cinnamomum cassia bark at different harvesting times
Source: BMC Plant Biol. 2024 Feb 2;24:84. doi: 10.1186/s12870-024-04754-w (PMC10835945; doi:10.1186/s12870-024-04754-w)
Supplement: Supplementary file 1 — Additional file 1: Supplementary Fig. S1. Differentially accumulated volatiles (DAVs) in different harvesting times. Supplementary Fig. S2. The venn diagrams of DAVs among four comparisons. Supplementary Fig. S3. K-means clustering analysis of the DAVs. Supplementary Fig. S4. Significantly enriched KEGG pathways of DEGs. [file 12870_2024_4754_MOESM1_ESM.pdf]

Supplementary Figures

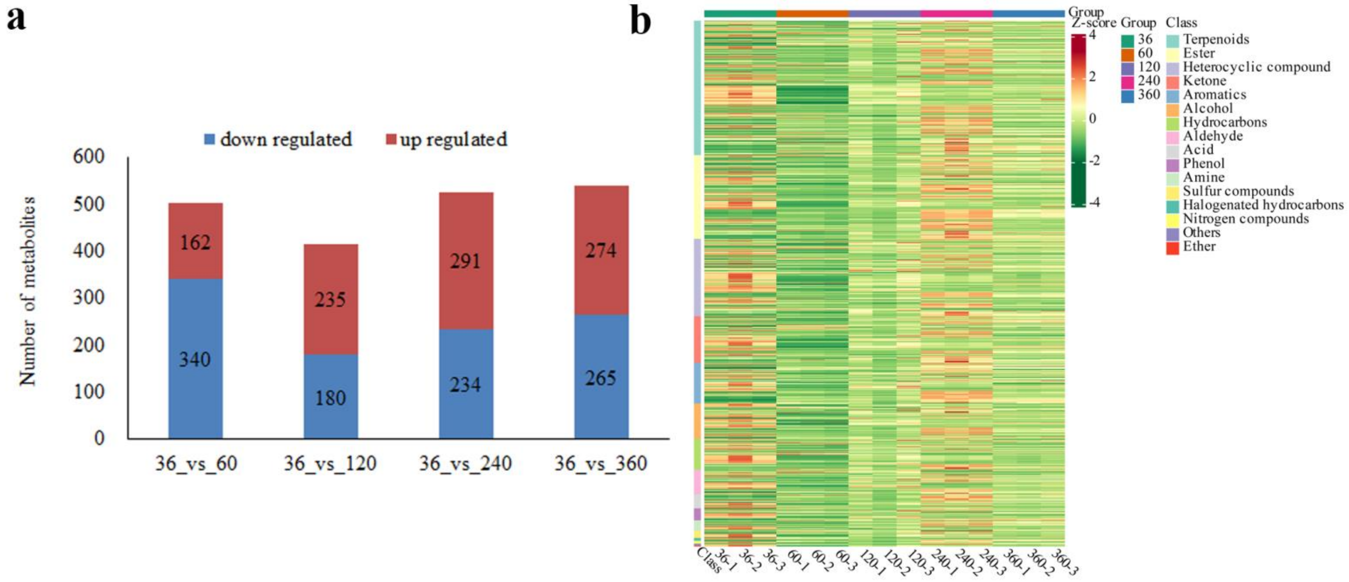

Supplementary Fig. S1. Differentially accumulated volatiles (DAVs) in different harvesting times. (a) The up-regulated and down-regulated DAVs in four different pairwise comparisons. (b) The DAVs and class in different harvesting times.

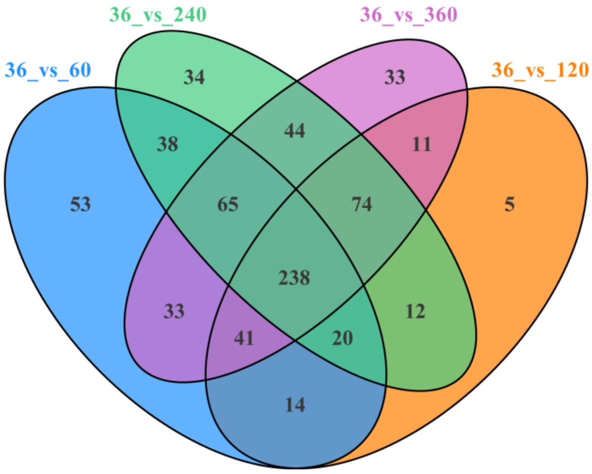

Supplementary Fig. S2. The venn diagrams of DAVs among four comparisons.

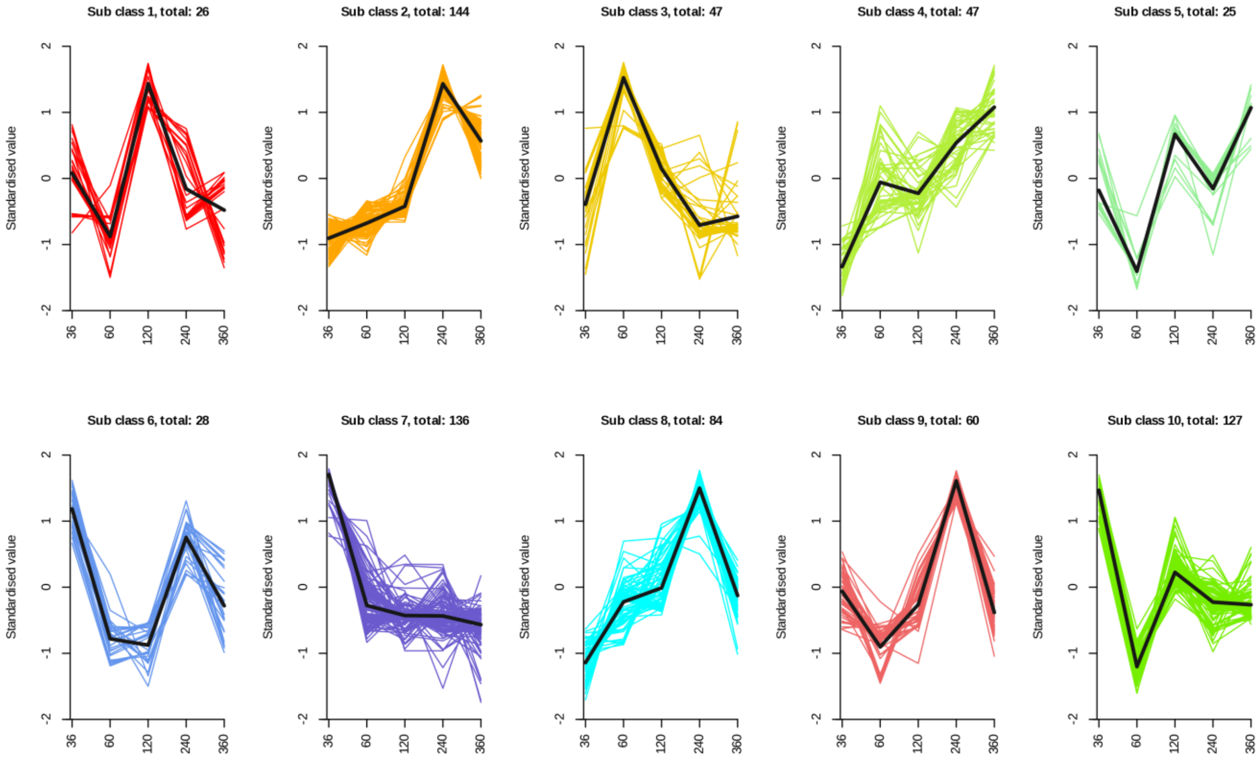

Supplementary Fig. S3. K-means clustering analysis of the DAVs

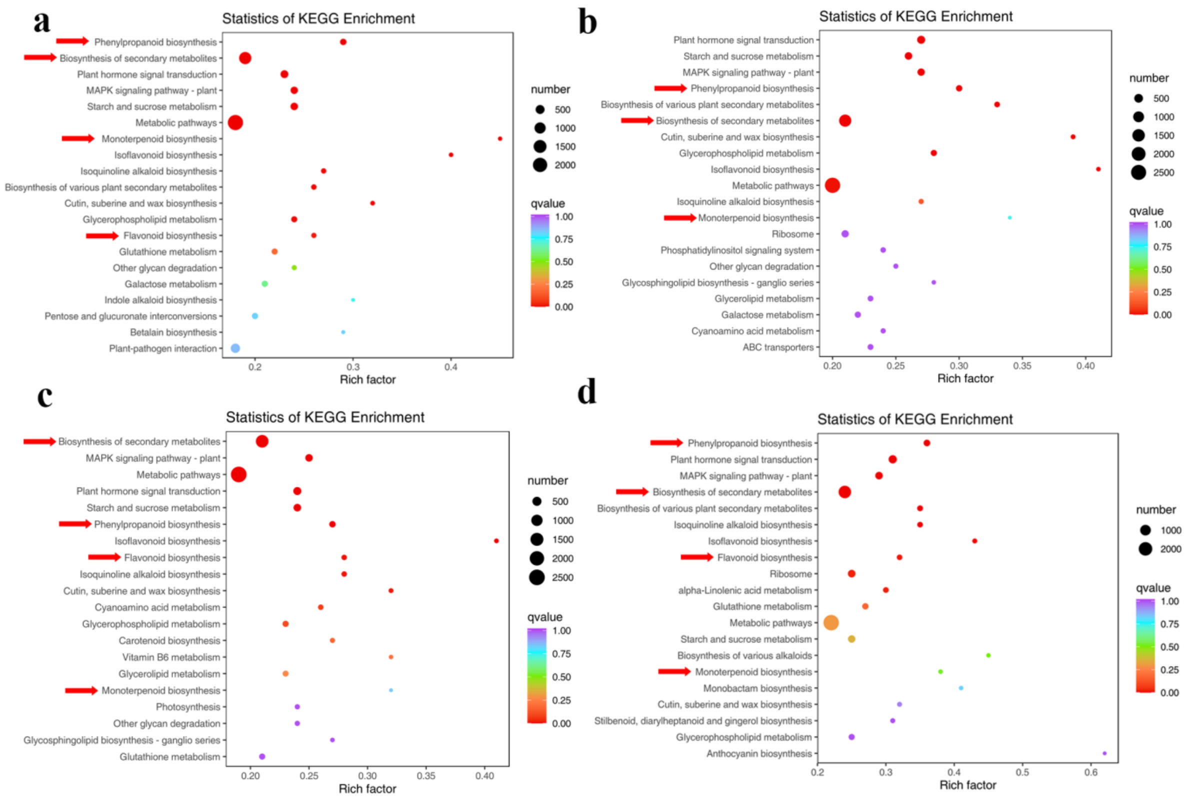

Supplementary Fig. S4. Significantly enriched KEGG pathways of DEGs. (a) 36-vs-60; (b) 36-vs-120; (c) 36-vs-240; (d) 36-vs-360.Top 20 significantly enriched KEGG pathways at pairwise comparisons. The Y-axis on the left represents KEGG pathways, and the X-axis indicates the "enrich factor" represented by the ratio of DEGs numbers to total annotated gene numbers of each pathway. Lower *q*-values are shown darker in the red circle. The area of a circle represents DEGs number. The KEGG pathways associated with terpenoid and phenylpropanoid biosynthesis marked with the red arrows.
